# Client’s Experiences Using a Location-Based Technology ICT System during Gambling Treatments’ Crucial Components: A Qualitative Study

**DOI:** 10.3390/ijerph19073769

**Published:** 2022-03-22

**Authors:** Laura Diaz-Sanahuja, Ignacio Miralles, Carlos Granell, Adriana Mira, Alberto González-Pérez, Sven Casteleyn, Azucena García-Palacios, Juana Bretón-López

**Affiliations:** 1 Department of Basic Psychology, Clinical and Psychobiology, Universitat Jaume I, 12071 Castellon, Spain; azucena@uji.es (A.G.-P.); breton@uji.es (J.B.-L.); 2 Department of Computer Languages and Systems, Universitat Jaume I, 12071 Castellon, Spain; mirallei@uji.es (I.M.); carlos.granell@uji.es (C.G.); alberto.gonzalez@uji.es (A.G.-P.); sven.casteleyn@uji.es (S.C.); 3 Department of Personality, Evaluation and Psychological Treatment, Valencia University (UV), 46010 Valencia, Spain; miraa@uji.es; 4 CIBER de Fisiopatología de la Obesidad y Nutrición (CIBEROBN), Instituto de Salud Carlos III, 28029 Madrid, Spain

**Keywords:** gambling disorder, control stimuli, exposure with response prevention, location-based technologies, satisfaction

## Abstract

Cognitive Behavioral Therapy is the treatment of choice for Gambling Disorder (GD), with stimulus control (SC) and exposure with response prevention (ERP) being its two core components. Despite their efficacy, SC and ERP are not easy to deliver, so it is important to explore new ways to enhance patient compliance regarding SC and ERP. The aim of this study is to describe and assess the opinion of two patients diagnosed with problem gambling and GD that used the Symptoms app, a location-based ICT system, during SC and ERP. A consensual qualitative research study was conducted. We used a semi-structured interview, developed ad-hoc based on the Expectation and Satisfaction Scale and System Usability Scale. A total of 20 categories were identified within six domains: usefulness, improvements, recommendation to other people, safety, usability, and opinion regarding the use of the app after completing the intervention. The patients considered the app to be useful during the SC and ERP components and emphasized that feeling observed and supported at any given time helped them avoid lapses. This work can offer a starting point that opens up new research paths regarding psychological interventions for gambling disorder, such as assessing whether location-based ICT tools enhance commitment rates.

## 1. Introduction

Gambling Disorder (GD) is classified as a non-substance-related disorder. GD involves repeated problematic gambling behavior that results in distress and significant problems. Individuals with GD have a lack of control over their behavior and, in spite of trying to stop gambling several times, they are unsuccessful. Individuals with GD have frequent thoughts about gambling, feel irritable when they cannot gamble, and need to increase the amount of money they gamble to accomplish the desired feeling of excitement. When they lose money, they often gamble again to “chase losses” and usually lie about the extent of their involvement. Due to these features, different areas of their life can be impaired (e.g., job, relationships, education). In addition to this complex symptomatology, GD has high comorbidity with other psychological disorders, with mood, anxiety, and substance use disorders being most prevalent [1,2,3,4].

GD is emerging as a relevant public health problem. In Spain, a recent study states that the prevalence of pathological gambling is 0.72% [5], higher than that indicated in DSM-5 (0.2–0.3%) [6]; a previous representative survey performed in a Spanish region using NODS [7] also indicates a prevalence of 0.3%. A study carried out by the Directorate General for the Regulation of Gambling [8] shows that, according to the NODS criteria, 24.3% of the studied population (*n* = 6816) are non-gamblers, 69.4% non-risk gamblers, 4.4% at-risk gamblers, 1% problem gamblers, and 0.9% pathological gamblers (PG) at any time in their life.

The first-line treatment for GD is Cognitive-behavioral therapy (CBT) [9,10,11,12], which has been further reinforced by systematic review and meta-analysis efficacy studies that report important and long-lasting improvements [13,14]. The core features of CBT for gambling disorder are stimulus control (SC) and exposure with response prevention (ERP) to gambling opportunities and cues. SC aims to prevent gambling behaviors, and ERP confronts the patient with an overwhelming urge with the purpose of habituating or extinguishing it [15,16,17].

SC is introduced initially to avoid gambling cues and establish an abstinence period. Afterwards, ERP is incorporated to achieve the habituation process of the urge to gamble considering the presence of a particular emotional reaction or gambling related stimulus. Both components are well established [18,19,20]. Despite the efficacy of CBT, SC and ERP components are not easy to deliver, and they are hard for the patients. There are inherent difficulties related to commitment during SC for those people who suffer GD, and high attrition rates and relapses are generally present [21]. This leads to a complex administration of treatment for patients diagnosed with GD and, therefore, it is important to enhance key therapeutic components and their motivational features.

Media-based tools have contributed to the development of new strategy designs to target psychological disorders. New communication technologies are not just tools, they are part of our culture [22]. In this sense, new technologies are embedded in everyday life and must be treated as natural elements of post-modern society [23,24]. Post-modern culture implies a deep and updated study of human interaction with new technologies, since, according to meta-analysis studies, they pose an important and urgent challenge [25,26]. In recent years, there has been increased interest in using technology-associated psychological interventions as a form of treatment for psychological disorders, including GD. Several studies have been conducted to improve the effectiveness of psychological treatments or clinical utility. The first promising technology was virtual reality (VR), which emerged as a viable and effective tool for psychological disorders, reporting efficacy in the treatment of GD and the ERP component [27,28,29,30]. Furthermore, media development has led to the use of the internet to deliver CBT, obtaining adequate results in randomized control trials (RCT) [31,32,33,34,35,36,37,38,39]. The efficacy of these self-guided treatments has been confirmed in many countries by scoping and systematic reviews or meta-analyses [14,40,41,42,43].

Information and Communication Technologies (ICT) systems for psychological intervention and their clinical effectiveness have been empirically tested. The issue of acceptability of technology-guided treatment is also important when considering whether or not the use of ICT systems will affect therapeutic adherence and clinical outcomes [44]. Acceptability is described as the degree to which users are satisfied or at ease with the service and willing to use it [45], and it is considered as an important influence in the perception of the treatment as fair and reasonable, appropriate, and non-intrusive in addressing a problem [46,47]. In the Technology Appraisal Guidance from the United Kingdom, to properly assess the intervention acceptability is considered a priority, and expectations, satisfaction, and usability are mentioned as variables linked to it. This, in turn, makes these variables crucial features in psychotherapy results [48]. Several studies have focused on expectations and satisfaction with different ICT systems. For instance, VR or internet-based systems for delivering psychological treatments [49,50,51], including for gambling disorder [31,32], have reported high satisfaction with the exposure component mediated by technologies. Studying acceptability and usability of an ICT system must take into account a very specific conceptualization within the user experience. With the aim of enhancing ICT system development, the ease of use of a product by a specific user should be explored with clearly defined context and goals. Even though few studies have addressed this topic [46,52,53], the aforementioned ICT system-driven examples have shown to be well-supported regarding acceptability.

Other tools included under ICT systems are mobile phone and smartphone apps. The large increase in mobile phones and smartphones over the years [54] offers additional and largely unexplored advantages for implementing psychological treatments for different mental disorders with the support of these technologies [55,56]. Hawker, Markouris, Youssef & Dowling [57] conducted a single-arm study that supports the acceptability, feasibility and preliminary efficacy of an app-delivered EMI for craving management in people with gambling problems. The app’s EMI feature recommends using 12 urge-curb tips or exercises that take 1 to 5 min to complete. The content is related to psychoeducation, relaxation techniques, and mindfulness (e.g., about my urge, delay and distract, and urge surfing). Smartphone apps have also been demonstrated to be feasible and acceptable as CBT adjunctive components to enhance homework completion in people suffering from a gambling disorder (e.g., decisional balance exercise, functional analysis of gambling behavior, development of healthy alternatives to gambling, problem-solving, and relapse prevention exercises) [58]. Moreover, a randomized controlled trial in which a self-help CBT program was combined with a messaging app showed promising results for overcoming the high dropout rate of unguided internet-based interventions for gambling disorders. Every day at 9 pm, participants in the intervention group received monitoring, personalized feedback, and messages based on CBT. Only 6.7% of the participants dropped out at follow-up and 77% continued participating during the trial period [59]. Recent RCT protocol studies include apps for assessing and delivering interventions for gambling problems [60,61]. It is important to improve the quality of psychological programs considering smartphone apps. Smartphone portability could be very useful in a variety of feared situations in which GD symptoms occur, and to enhance therapeutic components and adherence. A subset of ICT corresponds to location-based technologies (LBT-based ICT system) with location tracking and personalized feedback towards the patient based on their position. Smartphone apps that use LBT-based ICT systems could enhance key therapeutic components in specific disorders and could signify a starting point to initiate and sustain the behavior required during SC and ERP components. Consequently, this could lead to improvement in the patients suffering gambling problems by maximizing their motivation and commitment. Although LBT-based ICT systems could become a tool when prescribing some therapeutic components, it will be upon the patient to carry out the tasks on his/her own in several situations where gambling happens, both in offline and online gambling. Some advantages of using mobile devices with LBT-based ICT systems include ensuring that patients are committed to the SC and that they remain in the exposure situation for the necessary time to fulfill the ERP component goals.

In previous studies, when carrying out the treatment, the context and LBT-based ICT system have been considered and treated as a variable. In the study of Addepally & Purkayastha [62], the authors studied using a mobile application (app) that monitors the location of depressed people. The app detected if the depressed patients were in less-crowded areas (a common trait in depressed individuals according to the study) and, if affirmative, the patient would receive therapeutic strategies and self-help assessment through notifications. In a case report study [63], a patient suffering from obsessive-compulsive disorder was allowed to configure alarms for when she remained for an extended time period in the same place using a location tracking app. In a case study carried out by our research team, an LBT-based ICT system was used during the in vivo exposure (IVE) component in the Unified Protocol treatment of a 47-year-old patient with Panic Disorder and agoraphobia. The focus of the study was to enhance key therapeutic components during in vivo exposure and the patient reported positive expectations, high satisfaction scores, and an overall satisfactory experience [64]. Another tool was developed in Auckland (New Zealand) to treat gambling disorder. This tool, called the SPGETTI app, consists of two functions. On one hand, it supports relapse prevention by sending two notifications a day with content based on the Marlatt’s relapse prevention cognitive behavior theory; and on the other hand, it aims to help reduce harmful gambling, specifically the use of electronic gaming machines. The app uses geo-positioning technology (GPS) that recognizes when one is near places that have pokie machines and sends a few small messages to help the individual stick to their goals. This app needs an internet connection to send these messages and it is necessary to previously establish and configure general gambling zones. A prospective cohort study was conducted to explore the impact of the SPGETTI app (National Institute for Health Innovation (NIHI and University of Auckland, Auckland, New Zealand). Data were analyzed qualitatively using an inductive approach and notification messages were reported as positive in terms of how they supported gamblers to quit or reduce their harmful gambling [65]. However, to the best of our knowledge, there are no reports that account for an LBT-based ICT system in a smartphone application for the treatment of GD during SC and ERP, which allows its configuration to be tailored to each patient, be updated, and does not need internet connection to send notifications. The main aim of this study was to describe the use of LBT-based ICT systems in SC and ERP components during the treatment of two patients diagnosed with GD, and to assess the patients’ opinion about this LBT-based ICT system and the preliminary platform usability through qualitative analysis.

## 2. Materials and Methods

### 2.1. Patients

Patient 1 was a 28-year-old male with higher education who worked in a family company and was diagnosed with problematic gambling according to the Spanish-language version of the National Opinion Research Center DSM-IV Screen for Gambling Problems (NODS) [7]. He had not previously received treatment for this problem. He lived with his parents and had been in a relationship with his girlfriend for over two years. He placed bets on sports, especially soccer, and couldn’t remember exactly when the betting started, although he thinks that it probably started more than five years ago when he and his friends used to bet on soccer matches as a way of entertainment. During the last year before the treatment, he started to bet alone and spent most of his free time betting. The patient had no debts, but he recognized that he spent money uncontrollably during his gambling sessions. His main thoughts were focused on his sports betting skills and “the possibility of winning a lot of money through gambling”. He described himself as a competitive person who likes to win. He didn’t identify specific emotions linked to gambling but indicated feeling a kind of thrill while gambling linked to the possibility of winning money. He started to have significant problems, especially severe arguments with his girlfriend, because of the time he spent betting or thinking about it. It was then when he perceived gambling as a situation that was out of control and decided to request psychological help. He also decided to talk to his parents to inform them about the situation and received everyone’s support. At the beginning of the treatment, no co-therapist was involved, given that the patient was not currently gambling, the patient had not acquired any debts and had reported his gambling situation to his relatives. His girlfriend’s contact information (with her consent) was noted in case it was needed at any time during therapy. There was no substance abuse, and the patient wasn’t receiving pharmacologic treatment at the time. The patient mentioned having some social problems, probably linked to the social anxiety that can appear in those who are willing to receive psychological treatment once a gambling problem is addressed. When the patient attended therapy, he was trying to stop gambling.

Patient 2 was a 46-year-old single male with basic studies who was employed at a company. He met the diagnostic criteria for pathological gambling according to the Spanish-language version of the National Opinion Research Center DSM-IV Screen for Gambling Problems (NODS) [7]. He had not previously received treatment for this problem. He mostly played slot machines and additionally placed bets on different types of gambling. He started to play in 2009 as a way of entertainment, coinciding with the acquisition of a credit for the construction of his new house. Soon he started to think about the possibility of gambling as a way of winning money to pay for some of his expenses. He started to play more during the day, between 10 min to 3 h, changing the places where he played but with 3–4 favorite places. One day he spent more than 1000 euros playing slot machines and he progressively ran up important debts of approximately 23,000 euros from credit banks, friends and his work company. The main thoughts stated by the patient were “Now I’m going to win and I will be able to recover the loss” or “today I am going to be lucky”. Even when trying to stop his gambling behavior, he never succeeded. The patient identified some negative emotions strongly linked to his gambling behavior such as feeling alone. His sister and brother knew about the situation, and his brother was the person involved in the therapy process as a co-therapist. There was no substance abuse and no other psychological diagnosis. The patient was not receiving pharmacologic treatment at the time. As the patient’s positive characteristics and strengths, it is important to note that he was very dynamic, with significant social support and a wide range of pleasure activities which he used to practice on a daily basis. When the patient attended therapy, he was trying once again to stop gambling after significant economic and family problems.

### 2.2. Therapist

The therapist (J.B.) was a member of our research team with more than ten years of experience in the therapeutic field, including both psychological intervention and technology-mediated therapy.

### 2.3. Measures

Several common psychiatric testing measures were used to establish the diagnosis and to evaluate the effects of the intervention. However, given that the component under study were SC and ERP by location-based technologies (SC + LBT-based ICT system; ERP + LBT-based ICT system), only the measures related to both components are presented in this article (with the exception of the diagnosis measures).

#### 2.3.1. Diagnosis Measures and Measures for the Target Behaviors

Primary Outcome Measures:

NORC DSM-IV Screen for Gambling Problems (NODS) [7,66]. The NODS is a hierarchically structured 17-item screen that is designed to assess at-risk, problem, and pathological gambling. It refers to the gambling experience both throughout the person’s life and in the last year, with the alternatives being dichotomous (Yes/No). The total score ranges from 0 to 10 (1–2 affirmative items correspond with at-risk gambling; 3–4 items with problem gambling; and 6 or more with pathological gambling). The data obtained on specificity and sensitivity is good, its test-retest reliability is 0.98, and its validity is excellent considering that it corresponds strictly to the DSM-IV criteria.

Target behavior scales (adapted from Marks & Mathews) [67] were used to identify problem situations because of gambling. The target behaviors were defined as the behaviors linked to gambling and creating substantial impairment in the patient’s daily life. The patients rated the level in terms of the overwhelming urge or craving state prior to engaging in the specific gambling behavior (0 = nothing; 10 = maximum).

#### 2.3.2. Measures for Expectations and Satisfaction with the LBT-Based ICT System

The Expectation and satisfaction scale (adapted from Borkovec & Nau) [68] regarding the SC/ERP + LBT-based ICT system component was used previously in our research team in other ICT contexts [50,69], including pathological gambling [28]. This questionnaire was used to measure patient’s expectations, before, and satisfaction after, the SC/ERP + LBT-based ICT system component. The questionnaire includes six items: how logical the SC/ERP + LBT-based ICT system component seemed; to what extent it could satisfy the patient; whether the patient would recommend this component treatment to others; whether it would be useful in treating other problems; the component’s usefulness for the patient’s problem, and to what extent it could be invasive. This last item was considered a key factor in assessing the LBT-based ICT system during SC/ERP. Due to the main characteristics of the LBT-based ICT system, it was important to assess any disruption, annoyance or intrusion on the patient’s privacy caused by the LBT-based ICT system. Both parts (expectations and satisfaction) ranged from 0 to 10, being 0 = “not at all” and 10 = “very much”.

#### 2.3.3. Measures for Acceptability and Usability of the LBT-based ICT System

The System Usability Scale [70] is one of the most used tools for assessing perceived usability [51]. It consists of ten items, half written in a direct style and the other half in an inverse style. A five-point scale is used for rating the level of agreement, from 1 (strongly disagree) to 5 (strongly agree). This scale has a score contribution of the scale position minus 1 for the items 1, 3, 5, 7 and 9, and 5 minus the scale position for the items 2, 4, 6, 8 and 10. A formula is used to calculate the score as a percentage scale from 0 to 100.

#### 2.3.4. LBT-Based ICT System Qualitative Interview

This is a semi-structured interview developed ad-hoc based on the Expectation and Satisfaction Scale (adapted from Borkovec & Nau) [68] and the System Usability Scale [70] (See Supplementary Materials). It was developed following the principles specified in the CQR guidelines [71]. The primary team (L.D.; A.M.) discussed interview construction, then elaborated the questions, and the professor also did this separately (J.B.). Finally, agreement was reached when figuring out the number of questions. This included six open-ended questions that assess: satisfaction using the LBT-based ICT system; why they would recommend it to other people with gambling problems; utility; intrusiveness due to aspects of threat to confidentiality when using this technology; aspects of the tool that make it easier and/or more difficult to use; Likert scale measuring to what extent it could be helpful beyond treatment completion to help cope with gambling problems (from 0 “none” to 10 “very much”), and the reasons behind the score they give.

### 2.4. Treatment

The patients received a face-to-face intervention based on cognitive behavioral treatment (CBT) comprised of eight sequential therapeutic modules: motivation for change; psychoeducation; stimulus control (e.g., self-prohibition and blocking of usual gambling) and responsible return of debts (in the case of patient 2); cognitive restructuring; emotion regulation; planning of significant activities; coping skills and exposure with response prevention, and relapse prevention. The sessions included in each module were delivered weekly in around 1-h sessions. The SC + LBT-based ICT system and ERP + LBT-based ICT system components were carried out during the stimulus control and exposure with response prevention modules, respectively, keeping the structure of the aforementioned CBT-based intervention and including the LBT-based ICT system, which allowed the patient to receive personalized messages during the treatment process and specifically during the SC and ERP components. That is, during the exposure with response prevention modules, the patients were exposed to their main target behavior and used the LBT-based ICT system included in the study. The program’s content, including the eight modules with objectives and contents, can be seen in Table 1. All of these modules exhibit a similar structure: a therapeutic content part presented with text; exercises and activities; a brief summary of the module, and tasks to complete before continuing through to the following modules.

### 2.5. System Description

For the present study the Symptoms platform was used, adapted to the gambling disorder pathology considered for this work. The full tool has been described in previous studies [64].

**Figure 1:**
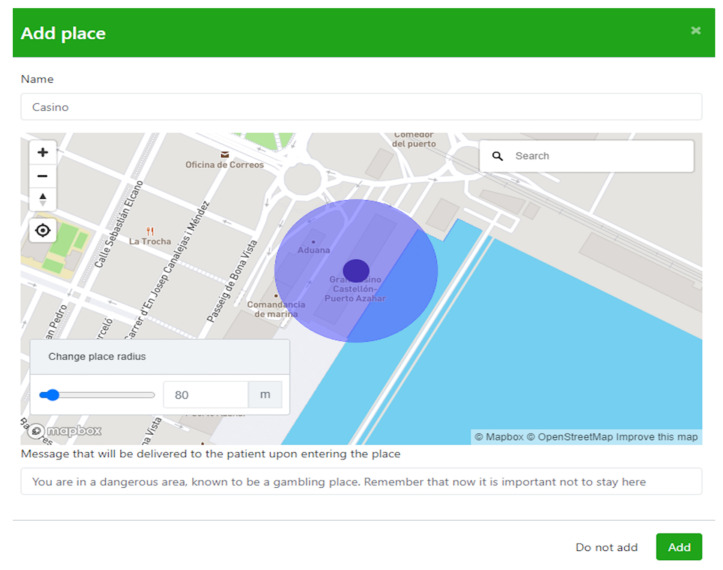
Smartphone app configuration during the SC therapeutic component.

**Figure 2:**
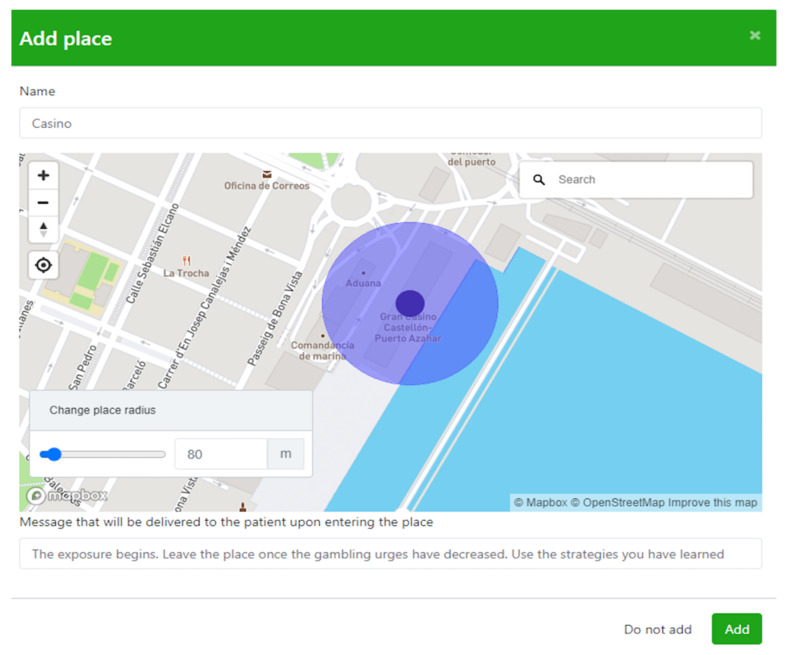
Smartphone app configuration during the ERP therapeutic component.

Symptoms is a technological platform that allows therapists to build, using the Symptoms Web application, an Ecological Momentary Intervention (EMI) smartphone app customized to the patient’s needs. For each patient, the therapist is able to indicate the relevant places for treatment and corresponding contents (e.g., personalized messages) to be delivered when the patient is in a particular place. Once the smartphone app is configured, patients install it on their smartphone and the app starts to monitor their movements on a regular basis (e.g., every five minutes). As soon as patients approach one of the relevant places, the app detects it through the LBT-based ICT system and reacts by delivering the associated content as indicated by the therapist depending on the therapeutic component patients are receiving, SC (Figure 1) and ERP (Figure 2). Finally, the therapist is able to check and evaluate the patient’s progress in a Web application to which the smartphone app communicates relevant data (whether or not patients have gone to the indicated places or if they have viewed the delivered messages, for example).

The Symptoms platform was designed to be flexible and configurable for different disorders, e.g., it has previously been used for agoraphobia as well [64]. In this study the platform was oriented to pathological gambling; therefore, the identified places and delivered information to patients were contextualized to gambling behaviors.

### 2.6. Design

This study follows a qualitative research method to evaluate the experience of two participants after using an LBT-based ICT system during SC and ERP. Specifically, the qualitative methodology used corresponds to the Consensual Qualitative Research (CQR) [67] based on the grounded theory. Both methodologies collect data using open-ended questions and conclusions from these data are reached through an inductive process. To report the study, the consolidated criteria for reporting qualitative research (COREQ) was followed [72].

### 2.7. Data Analysis

The narrative content from the qualitative interview was analyzed following the CQR process. For this purpose, a primary team was formed which consisted of a PhD student (L.D.) and a PhD graduate (A.M.), both females, with around five years of clinical training experience and also experience with the use of ICT for delivering interventions. Both attended a qualitative research course which addressed CQR. They had no previous therapeutic contact with the participants, and served as judges in the coding process. For the qualitative data codification, domains, core ideas, and categories were established separately. Domains consist of topic areas, core ideas are abstracts or brief summaries from the participant’s dialog, and categories correspond to consistencies in the core ideas within the domains. The primary team reached a consensus regarding this codification through discussion, and if there were discrepancies, an auditor, female full professor (A.G.-P.), expert in the field of psychopathology treatments, would help solve them. The auditor also checked the codification and gave comments to the primary team, which continually went back to the raw data to make sure that the results and conclusions were accurate and based on the data. The consensus process relies on mutual respect and equal involvement [73].

In addition to the qualitative interview, results for assessing patient’s opinion about the use of the LBT-based ICT system for delivering SC and ERP therapeutic components, raw scores regarding expectation, and both the satisfaction and usability scales are reported. Even though it was not a goal of the present study, scores regarding the overwhelming urge in the target behavior are reported at the 1, 3, 6 and 12-month follow-up periods to show improvements.

### 2.8. Procedure

The patients asked for help at the Jaume I University Anxiety Disorders Clinic, Spain. First, they underwent a face-to-face screening assessment and, having met the inclusion criteria, they signed a consent form to participate in the present study. Inclusion criteria included the following: (a) meeting the gambling disorder diagnostic criteria (GD or problematic gambling), and (b) providing written, informed consent and also consenting to being recorded. Exclusion criteria included: (a) suffering from a severe comorbid mental disorder (schizophrenia, bipolar disorder, and alcohol and/or substance dependence disorder); (b) medical disease/condition that prevents the participant from carrying out the psychological treatment, and (c) receiving another psychological treatment during the study. The assessment consisted of around two 60-min face-to-face sessions to evaluate the diagnosis and establish the target behaviors related to the gambling behavior. The NODS was carried out in the first session and the second session was used to fill other self-report measures and establish the patient’s target behaviors. Additional measures linked to the treatment were carried out in the context of the full therapy. Given that the component under study was the SC/ERP + LBT-based ICT system, only the measures related to this component are presented in this article and emphasized in the measures section. Following the assessment, and before starting the treatment, the therapist explained the basis of the treatment and the use of the LBT-based ICT system to support the mentioned therapeutic components. The patients agreed to take part in the research. The expectation with the SC/ERP + LBT-based ICT system components was evaluated by the patients before the treatment was conducted.

When the consent form for participating in the research and the consent form to use the LBT-based ICT system were filled out properly, the treatment started. Therapist and patient created the core situations linked to the gambling behavior and consequently established their target behaviors, which were assessed before each treatment session with the target behavior scale. The app was installed on their own smartphones, and the locations were positioned by the LBT-based ICT system and configured to receive the notification when the patient arrived at these core places during SC and ERP. During the SC module, every time the patient arrived at one of the core places, they received a notification with the particular message configured previously by the therapist “You are in a risk area because it was a place of gambling for you. Remember that it is now important not to stay here”. After the first use of the app, following the recommendations given by the theoretical framework of ICT usability [50,51], the System Usability Scale was filled out by the patients. During the ERP module, every time the patient arrived at one of the core places during the exposure tasks, he received a notification with the particular message configured previously by the therapist “You are in a relevant place, the exposure begins. If there is an urge to gamble, use the strategies you have learned and leave the place when the urge has decreased”. Following all the exposure tasks and after treatment, the satisfaction with the SC/ERP + LBT-based ICT system components was measured by the patients with the self-report measures, and the LBT-based ICT system component usability assessment. Finally, a qualitative interview was conducted at a 12-month follow up via videoconference. The interview was led by L.D., who had not been involved in the intervention process and lasted between 30 and 40 min. It was audio-recorded and later transcribed by two independent researchers (L.D and A.M). Once a single unified version of the transcription was obtained, the narrative content was analyzed independently to establish domains, core ideas, and categories. The primary team came together to discuss ideas and reach a consensus regarding codification. The auditor checked the work of the primary team and gave feedback. The primary team then assessed this feedback through a consensual process to establish a single unified version.

## 3. Results

Patient 1 carried out the weekly treatment for a period of 4 months and patient 2 carried out 5 months of weekly treatment during the time of the study.

### 3.1. Target Behaviors

**Figure 3:**
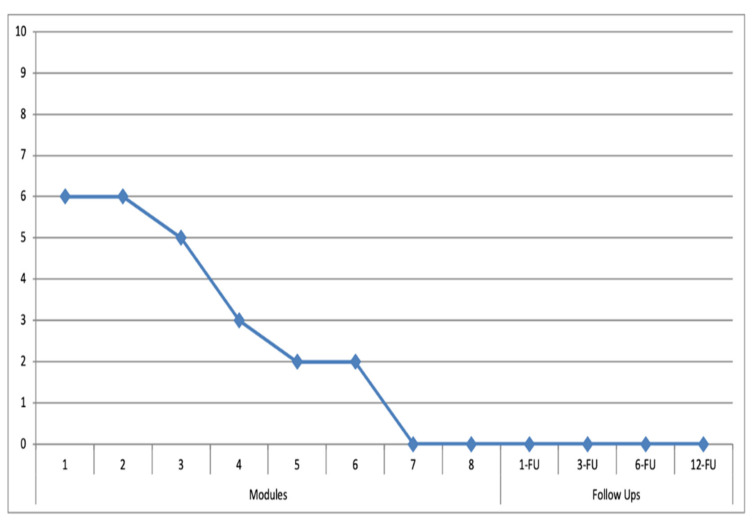
Excitement and urge (0–10) to follow a strategy and the possibility of winning money through sports betting for patient 1. (Modules = 1 to 8 session treatment; 1-FU, 3-FU, 6-FU, 12-FU: 1-, 3-, 6-, 12-month follow-ups).

**Figure 4:**
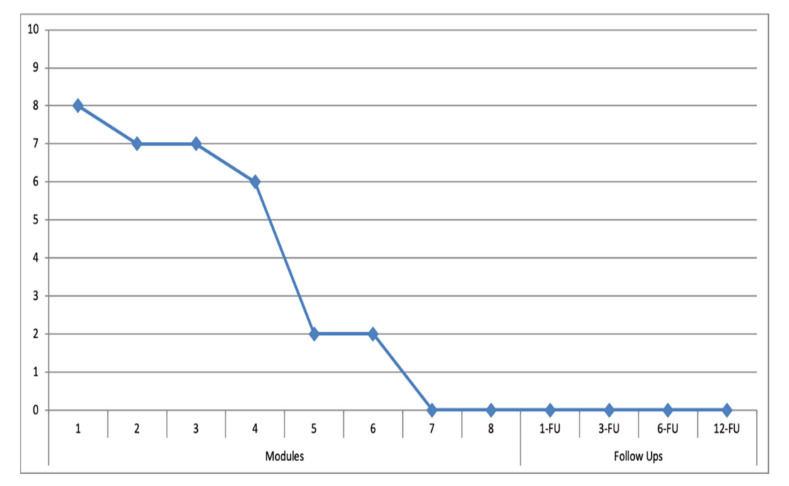
Overwhelming urge to play slot machines (0–10) and distrust regarding his own capacity to resist it for patient 2. (Modules = 1 to 8 session treatment; 1-FU, 3-FU, 6-FU, 12-FU: 1-, 3-, 6-, 12-month follow-ups).

In Figure 3 and Figure 4, the main target behaviors established by the patients and therapist regarding the gambling behavior, as well as the overwhelming urge ratings from the patients are shown. As shown in Figure 3 and Figure 4, they are “sports betting” (for patient 1) in which the patient highlights the “excitement and urge to follow a strategy and the possibility of winning money” and “slot machines” (for patient 2) where the patient emphasizes a “strong overwhelming urge to gamble and distrust regarding his own capacity to resist it”. The selected main target behaviors caused a certain (severe in the case of patient 2) level of impairment in different areas of the patients’ life. Once the treatment started, there were no relapses reported by the patient or the co-therapist. Even though it was not a goal of the present study, preliminary results regarding the overwhelming urge in the target behavior show an important score reduction in the scheduled situations, supported by the LBT-based ICT system during the treatment and were maintained at the 1, 3, 6 and 12-month follow-up periods.

### 3.2. Expectations and Satisfaction regarding the SC/ERP + LBT-Based ICT System Components

#### 3.2.1. Patient 1

**Figure 5:**
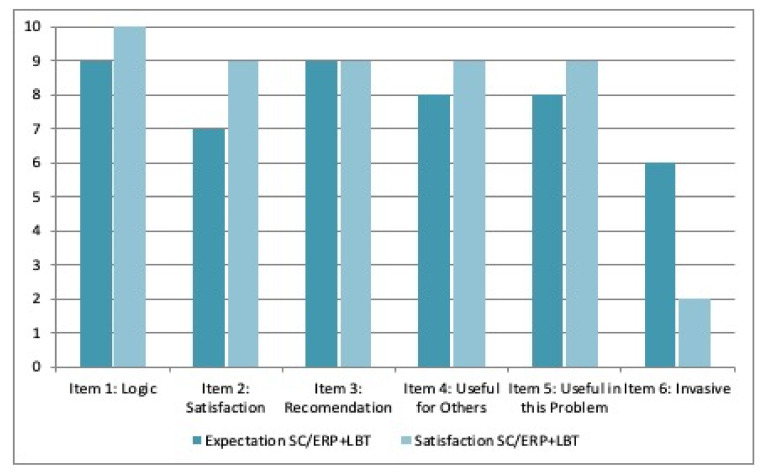
Expectation and satisfaction with the SC/ERP + LBT-based ICT system by patient 1.

Patient 1 reported high expectations before starting the treatment and a high satisfaction after receiving it. Specifically, the patient considered that it could be invasive in a moderate form before starting the treatment, but after the intervention, invasiveness was assessed as low (Figure 5).

The perceived usability score was high in both assessment moments (first app’s use and after treatment) and increased slightly after the intervention (Table 2). In particular, one of the important aspects that improved was confidence when using the system.

The overall value for satisfaction and usability was 87.5 points after the first use and 92.5 after treatment, which, according to the qualitative scale developed by Bangor, Kortum, & Miller [74], means that the system was within an acceptable range, with adjectives rating between “excellent” and “best imaginable”.

#### 3.2.2. Patient 2

**Figure 6:**
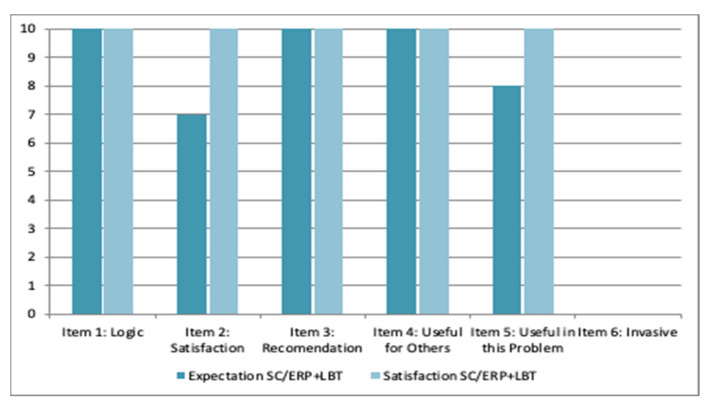
Expectation and satisfaction with the SC/ERP + LBT-based ICT system by patient 2.

Data about expectations at pre-intervention and the satisfaction after receiving it are reported in Figure 6. He reported high expectations before starting the treatment and high satisfaction after finishing it.

In addition, patient 2 reported a high perceived usability after the first use and after the intervention (Table 3). He did not consider it at all invasive, and the overall value for satisfaction and usability was 100 points for patient 2 in both assessment periods. This score is the maximum of the scale and, according to the qualitative scale developed by Bangor, Kortum, & Miller [74], it means that the system usability perceived for this participant was the “best imaginable”.

### 3.3. Qualitative Interview

Following the CQR methodology, the three main aspects to report correspond to domains, categories and core ideas. On the whole, six domains and twenty categories were found. Table 4 shows the results of the qualitative analysis.

#### 3.3.1. Domain 1: Usefulness

Patients indicated that the app was useful for different purposes throughout the intervention. We can divide them into six categories ordered according to their importance: vigilance; lapse/relapse prevention; stimuli control; accompaniment/protection; reduction of the lapse/relapse duration, and gambling urge habituation.

Vigilance: This category refers to the perception of being observed. This perception gives the patient and their families the confidence to be abstinent. For example, patients explained: “I think it can be useful, it is a warning, as if someone is observing me, thus I do not go into gambling-related places” (patient 1). “Knowing I am observed helps me to not give into the temptation, nor into the confidence of thinking ‘I have this problem, but since I am not being observed and no one knows what I am doing, I am going to bet again”; “I think that feeling that one is observed is an advantage, to be alert to not have a lapse, and it offers assurance to both me and my family” (patient 2).

Lapse/relapse prevention: This category is described as the ability to cope with gambling urges when risk situations are present. For example, patients mentioned: “The app is one more tool to cope with gambling urges when one is in risk situations. It is as if one had a ‘Jiminy Cricket’. If one’s conscience does not help to cope with gambling urges, the app is useful because it gives recommendations to avoid lapses” (patient 1). “It helped me cope with gambling urges and to deal with risk situations (i.e., if I was close to gambling venues). Also, to reassert I was on the right path and prevent lapses” (patient 2).

Stimuli control: This category corresponds to the prevention of finding oneself at risk situations such as a gambling-related venues. When the SC therapeutic component is applied, gambling urges are probably high, and it is important that patients avoid these types of places. They considered that the app helped them achieve this objective. For example, they reported: “It is a tool that stops oneself from going into gambling venues, and in case one continues to be close to these areas, the app sends the message again, which I think is positive” (patient 1). “It helps you to be aware that going to those places is not a good idea, that it is wrong and, therefore, you don’t go directly, or when you are getting closer, you decide to leave” (patient 2).

Accompaniment/protection: This category refers to the perception of being supported throughout the intervention, keeping oneself safe from gambling. For instance, patient 2 mentioned: “The app was part of my day to day, I carried it, and it protected me”; “For allergies I take a pill to avoid sneezing and it protects me, in the same way the application at that time was a protector against gambling”; “The app protects me from gambling as my environment (i.e., my friends or family) does”.

Reduction of the lapse/relapse duration: This category is described as the ability to shorten the time patients are gambling when a lapse is produced. For instance, patients mentioned: “It can even be useful when a person who did not respond to the first message and did not cope with gambling urges adequately goes into the gambling venue, because once inside, the message ‘you are making a mistake’ continues. It can help for limiting the time that one is making the mistake or relapse” (patient 1). “It would have helped me if I had had a relapse, because when one sees the message twice, one thinks ‘I’m going home to the safe zone’” (patient 2).

Gambling urges habituation: This category means that the app can help participants when the exposure with response prevention therapeutic component is introduced to reduce the intensity of the gambling urges. For instance, patient 1 mentioned: “When I had to stay in that situation until the gambling urges decreased, the app helped me achieve that goal”.

#### 3.3.2. Domain 2: Improvements

This domain refers to the improvements that could be considered when using the app. There are four categories related to it: adding places to the app by contrasting the information with the co-therapist; increasing the feedback to the therapist; therapist assistance at the risk situation of a lapse, and increase in the emotional impact of the messages.

Adding places to the app by contrasting the information with the co-therapist: Patients could deceive themselves and not give all the gambling sites when configuring the app. They could go to other gambling venues that have not been mentioned when configuring the app. So, a possible improvement is to contrast the information with the co-therapist regarding the different gambling venues patients used to go to. Although it is a measure that could improve the intervention efficacy, there is always the possibility of forging the answers, and sincerity is really important for therapy success. For example, patients said: “It can be tricky if one is not fully involved in the treatment. As one has to say where the gambling venues one used to go to bet are located, it is possible to go to a place not included on the list. I think it is difficult to improve this aspect” (patient 1). “I was the one who gave information about the gambling venues, and I was very sincere. However, maybe other people with gambling problems are not sincere. I consider it would be positive to involve other people in this moment of the therapy to contrast the information and also because they could add more gambling venues than the ones reported to the therapist. In my case, for example, it would be my brother” (patient 2).

Increasing the feedback to the therapist: This category indicates the importance of sending more specific information about the location of the patients to the therapists, such as the amount of time they spend at every site, that could be related with gambling activities. For example, patient 1 said: “Knowing how important it is that the treatment really goes well, I think it would be appropriate that the psychologist knows through the app how long the person has been at the sites. The reason is that a person may not be sincere and could say that they did not enter a gambling venue. However, if the therapist would have this information, it could be useful to contrast the information” “Although firstly the patient could consider it against their privacy, in the long term it would be positive and effective because it is another way to increase the control over patients and protect them”.

Increase of the emotional impact of the messages: This category refers to changing the type of message by introducing the negative consequences that gambling can cause in different spheres: social, personal, financial, economic, or work. For example, patient 1 said that it could be appropriate to include messages such as: “you are losing money”; “you are compromising the health of your family members or your possible future relationships”; “you are jeopardizing your family’s finances”. The patient explained that instead of only saying “you are in a risky place for gambling and you need to go”, it would be appropriate to include powerful messages like those based on anti-tobacco advertising campaigns or the National Department of Traffic (DGT). On the cigarette packets there are messages such as “you could get lung cancer”. According to this idea, it would be a stronger way to influence people to stop gambling when at a gambling venue.

Therapist assistance during a risky lapse situation: This category refers to the therapist receiving information about the patients to increase support when they are in a risky situation. For example, patient 1 mentioned: “The therapists could have access, or a warning could be sent to them in some way when the person was in the same place for a while, since it could mean that the patient may have been gambling at that place. And perhaps in the next session this could be discussed in therapy”; “If at that moment the psychologist could call the person or offer some kind of assistance, I think it would be even better of course, because that person would be treated during their moment of weakness. Maybe the patients could even explain how they felt or what was influencing them to gamble. But I think this would be more difficult because perhaps the psychologist is not available at that time and cannot call the patient at that moment”.

#### 3.3.3. Domain 3: Recommendation to Other People

This domain encompasses two categories related to recommending the app to other people suffering from gambling problems as well as to other people suffering from other psychological problems.

Extra support for other people suffering gambling problems: This category refers to the consideration that as the app provides patients additional support throughout the intervention to increase self-efficacy to cope with gambling urges and to prevent lapses, they would recommend its use to other people with similar problems. For example, they mentioned: “If I think it is useful for me, I would also recommend it to someone who is in a similar situation, because I think that those advantages it has will give them extra support that without using it they do not have” (Patient 1). “I would recommend it 100% because one feels accompanied, controlled and observed and it serves as support for increasing self-efficacy to cope with gambling urges and helps to avoid a lapse” (Patient 2).

Assistance in the treatment of other psychological problems: This category shows the perceived applicability of the tool for the treatment of other psychological problems such as substance use disorders (i.e., cocaine, marijuana, alcohol, etc.). For instance, patient 2 indicated: “It is a very positive tool, both for the treatment of gambling problems, as substance-related problems such as alcohol, cocaine or marijuana. In these cases, the app knows where one is located, and this can help a lot”.

#### 3.3.4. Domain 4: Safety

This domain includes two categories: intrusiveness and confidence. Intrusiveness is understood as the sensation of discomfort or insecurity due to aspects of threats to confidentiality using the technology; and confidence corresponds to the perception of calm and protection.

Patient 1 considered the app could be intrusive at the beginning, but after explaining the therapeutic purpose of its use, he considered it did not suppose any privacy problem and he felt confident using it for dealing with gambling problems. He mentioned: “I understand that maybe at first providing data of one’s location can cause a bit of discomfort, but not beyond that initial moment”; “As the objectives are therapeutic and these messages arrive and accomplish their function of guiding, you focus on what is important and it gives one the assurance that it can help”. Patient 2 indicated: “It is not intrusive, if it was intrusive I would not be following the recommendations the therapist gave me”; “In other type of apps sometimes one does not want to activate the location option, but in this case it was highly recommended and it helped me a lot”; “People who use the app do so because they need it, and they have to be willing to do what therapists recommend to deal with gambling problems, otherwise one will not get out of this problem”.

#### 3.3.5. Domain 5: Usability

This domain refers to the ease or difficulty of using the app, and includes two categories, referring to the usability at the moment of its installation and to its use throughout the intervention. Both patients considered the app to be easy to install and simple to use throughout the treatment process.

For example, regarding the installation category patients indicated: “The procedure to download the application was easy, it is similar to the one followed with other applications” (patient 1). “When installing the application, I did not have any complication, I followed the instructions and it was easy” (patient 2).

In terms of the usability throughout the intervention they mentioned: “During the treatment, the messages simply appeared if I was at the gambling-related places that I had indicated previously and that’s it, that’s why it seems easy to me” (patient 1). “The app is simple, once you have it installed and you have activated the location-based position it functions autonomously”; “If you want to listen to music, even though one has the Spotify app installed, one needs to access the app. However, this tool is always activated and functioning, one does not have to access it each time” (patient 2).

#### 3.3.6. Domain 6: Opinion for Using the App after Completing the Intervention

This domain illustrated to what extent they believe that using the app after completing the intervention could be useful for coping with gambling problems and the reasons behind their opinion. Three categories are distinguished:

Support to be abstinent: This category refers to the patients perceived usefulness to continue using the app after the intervention because it can help to cope with gambling urges and to be abstinent. For example, patients mentioned: “Because it does not suppose a privacy problem and the function is effective, I would have no problem to continue using it for as long as necessary. It is a reminder to be abstinent, so I consider this tool positive” (patient 1). “It can always help, even after finishing therapy because of the sensation of being observed and not tempted to bet again” (patient 2).

Severity of gambling-related symptomatology: This category represents the convenience of using the app even after treatment for those cases where gambling urges are still high and the self-efficacy to cope with them is low. The app could be used in more severe cases to remind them when they are at risk situations. For example, patient 1 mentioned: “Whether or not it is advisable to continue using the app would depend on the severity of the problem or the ability to cope with gambling urges. In my case, gambling urges at the end of the treatment were low, so it has been easier to control these situations and decide not to bet. However, to a person who has finished the treatment and still has a desire to bet, it can be useful”.

Updating: this category is related to the convenience of using the app after the intervention in order to be alert of risk situations and to be abstinent, but also updating the risky gambling-related places. Patient 2 mentioned: “It would serve to remain vigilant to risky situations, to be careful, to keep it in mind, to avoid lapses, but one would need to also update the sites because one can also change their routine”.

## 4. Discussion

This study analyzes clients’ experiences with the use of an LBT-based ICT system during SC and ERP therapeutic components. The results obtained in this qualitative study, including two participants’ experiences, show that the use of LBT-based ICT systems could be relevant for innovation in the treatment of gambling disorder with different types of severity. There seem to be several positive opinions about using the LBT-based ICT system for delivering SC and ERP therapeutic components. (1) It helps prevent being at risk situations such as gambling-related venues and achieve gambling urges habituation, respectively; it helps prevent lapses/relapses when risk situations are present, and in case one has a lapse it can help reduce the duration of gambling behavior; it is perceived as a tool that serves as accompaniment and protection, giving individuals the sensation that they are being observed and could increase the perceived confidence to be abstinent. Thus, it helped them to reassert that they were making adequate decisions for coping with their gambling problems. (2) It would be recommended for other people suffering gambling problems as extra support for coping with gambling urges and preventing lapses, as well as for the treatment of other psychological problems (e.g., substance related disorders); (3) It is recommended even after completing the intervention depending on the problem severity because it can support patients in their abstinence goals, but considering that it is necessary to update the previously configured gambling venues, which is possible because this app has this function. (4) The technology is well-accepted by the patients, showing positive expectations and high satisfaction. The app provides confidence considering the function it has for guiding during the intervention. These results are in line with those from Oakes, Rene & Lawn [75], a qualitative study which concluded that social support is considered an important aspect for preventing lapses because it provides a safety net that enables one to continue being abstinent when PG experience distress. The LBT-based ICT system could be useful for supporting patients throughout the intervention and after completing it. It could help increase adherence to treatment and reduce dropout rates. However, this information is only qualitative, and we need more research to reach conclusive findings. Patients considered it would be relevant to continue using the app after completing the treatment in higher severity cases because the app can support and help them cope with gambling urges and abstinence. According to Hodgins & el-Guebaly [76], a precipitating factor for gambling behavior could be the cessation of support on treatment follow-up. In addition, Jimenez-Murcia et al. [77] highlight the importance of incorporating interpersonal support in gambling disorder interventions to improve treatment outcomes, prevent relapses, and increase adherence. Relapse prevention strategies are relevant in dealing with high-risk situations. Gambling cravings and low confidence in one’s ability to resist a craving to gamble lead to gambling lapses and spending more money [78], so it would be convenient to implement tools for supporting patients after completing treatment to better manage cravings and avoid lapses. One convenient option to continue exploring would be to incorporate LBT-based ICT systems because they could help reduce lapses when patients find themselves in a high-risk situation or experience gambling urges during follow-ups.

The reported positive expectations and high satisfaction scores by the patients coincide with previous studies showing that ICT treatments are well-accepted [46,47,48,49,50,51], also in pathological gambling studies [31,32]. Specifically, LBT-based ICT systems have been well-assessed in a previous pilot study with another pathology, namely a case study of panic disorder, obtaining promising preliminary results [44]. These results with more advanced technology (LBT-based ICT system) are of high importance, since a positive relation between expectations and satisfaction with the ICT treatments and intervention efficacy have been found [79]. Consequently, it is important to continue improving the treatment by innovative tools that could have direct implications on effectiveness. An important aspect is to what extent the LBT-based ICT system considered for the study could be invasive. Patient 1 considered invasiveness as low, especially after the intervention, and patient 2 expected that the system would not be invasive at all. Due to the type of LBT-based ICT system considered in this work and the targeted disorder, the data on intrusiveness are especially relevant. The loss of privacy due to detailed information (location tracking) about the system’s usage being sent to the therapist could have created interference with the opinion about the system. However, this was not the case in this study and the LBT-based ICT system was not considered invasive or an interference in the fulfillment of the SC and ERP components. It did not pose problems in terms of privacy or insecurity regarding confidentiality when using this technology.

Finally, preliminary usability results reported the patient’s satisfactory experience with the system. According to the qualitative scale developed by Bangor, Kortum, & Miller [74], this means that the system could be within an acceptable range, with adjectives rating between “excellent” and “best imaginable”. Based on the technology acceptance model, these authors have suggested that one of the factors that can be related to the intention to use a product in the future is ease of use [80,81]. Therefore, usability, as an important attribute in the use of any technology [82], is a key prerequisite in the use of technology for psychological interventions. Given the point of view of patients, the Symptoms app was easy to install and to use throughout the intervention. Technology must be completely easy and clear from the beginning, otherwise, a slow learning curve or high frustration during use would affect the therapy and negatively impact the outcome of the intervention. Consequently, the usability of new ICT system approaches such us LBT-based ICT systems is decisive, and more sophisticated studies should try to ensure it.

Previous research has focused on the importance of using personalized feedback interventions for gambling disorders [83,84] and how it can show success as a low-cost intervention for reducing problematic behavior in addictions. The use of innovative ICT systems such as LBT-based ICT system subsets can be a first step towards a more effective treatment. Specifically, the development of particular LBT-based ICT system strategies as additional tools to guide the SC and ERP components could be useful in increasing overall functioning, enhancing the motivation and commitment of the patient with critical components of the therapy and reducing the abstinence violation, relapses and dropouts, especially important in pathological gambling [85]. However, more sophisticated studies are needed for this purpose.

From a technical point of view, and given the simplicity of the creation of the application, the adaptability of this tool is also presented as a positive feature. Changing the indicated places or the information delivered depending on the therapeutic component (SC or ERP, for example) can be done quickly and without requiring specific technical knowledge. This makes it easy to customize applications for different patients and therapeutic components. As was mentioned in previous studies [64], generating this type of in-situ intervention has always been somewhat complex, requiring time-consuming traditional methods or trusting the patient to carry out the indicated tasks. Thanks to this tool, this process has been streamlined, allowing therapists to focus their attention on the therapeutic content and delegating the monitoring and delivery of the materials to the mobile device. Future improvements of the tool have been discussed [64] and could have important utility in the context of gambling disorder. This is the case of including additional variables to improve the quality of the intervention, such as different messages arriving depending on the time (a message at the beginning of the ERP component, a message in the middle, and one at the end of this therapeutic component), or more complex content such as multimedia resources (images or videos) which can help during the exposure to the relevant target places for the gambling behavior. To extend the application of the tool beyond the intervention and include feedback in the form of questionnaires that are able to identify, for example, the overwhelming urge of the patient, could also be very useful. In addition, we should take into account the qualitative data obtained regarding the improvements domain. For instance, adding gambling venues to the app by contrasting the information with a co-therapist, increasing the feedback to the therapist in case patients agree, increasing the emotional impact of the messages when people with gambling problems have a lapse to help them stop gambling, and increasing therapist assistance during a risky lapse situation to prevent a relapse.

In summary, the idea is to maximize the use of smartphone applications with a high ecological value in the field of psychological treatments. Despite the rapid increase over recent years in the number of psychological interventions for various mental disorders using smartphone-based apps, a more innovative use of smartphones’ capabilities, such as sensing, alternative delivery paradigms, and advanced analytics, has not been explored in psychological treatments and, concretely, for gambling disorder [86].

This study has some shortcomings. The main one is that it is a qualitative study, so we cannot generate conclusive findings regarding acceptability and usability. However, our findings are related to client opinion and experiences using an LBT-based ICT system during the SC and ERP therapeutic components, and we show indicators regarding acceptability and ease of use by the patients in this study. We consider it to be relevant to start using LBT-based ICT systems during SC and ERP with people suffering from gambling problems in qualitative designs to understand preliminary satisfaction, usability, and acceptance. In other research fields, systems such as augmented reality (AR) were first used in case designs [87,88] followed by multiple baseline designs across-individuals [89], and randomized controlled trials [90]. The use of LBT-based ICT systems was also used for the treatment of other psychological disorders in studies with case designs [63,64]. Nevertheless, in order to increase confidence in the described SC/ERP + LBT-based ICT system, it would be necessary to apply this technology to larger samples in future robust studies with an experimental design that includes a control group. Thus, results should be considered with caution given that it is a qualitative study with all of the threats regarding internal and external validity that this implies [91,92]. In addition, the application at the moment only allows for a basic configuration (information in text format and places) as was mentioned before. However, despite these limitations, we believe that this study offers a starting point that opens up new paths for improving psychological interventions through the use of smartphone devices which could offer promising possibilities regarding the increase of treatment adherence.

## 5. Conclusions

Although SC and ERP are the two core components for the treatment of gambling disorder, and are evidenced-based, it is hard for patients to apply them, and high attrition rates and relapses are generally present. The Symptoms app uses location-based technology and sends personalized messages for enhancing these therapeutic components. Expectations, satisfaction, and usability regarding the Symptoms app reported by both patients were high. LBT-based ICT systems could be an important tool for increasing treatment adherence and commitment while delivering SC and ERP, and could improve the quality of interventions for GD and its efficacy. In this qualitative study, gambling urges decreased during the intervention, were maintained at low levels, and no relapse was produced during the 12 months’ follow-up period. Further studies with larger samples need to explore the effect of an LBT-based ICT system on the efficacy of psychological interventions for GD as well as the impact on adherence and commitment.

## Figures and Tables

**Table 1:** Treatment content.

Module	Objectives	Contents
M1. Motivation for change.	Providing information about the specific program and increasing motivation for change.	- Brief description of the content of each module. - Change stages in addictions. - Decisional balance. - Differentiation between lapse and relapse. - Establishment of general and specific objectives, and steps required to achieve these aims according to personal values.
M2. Psychoeducation.	Understanding gambling.	- Chance games’ characteristics. - Reasons for gambling. - Gambling stages. - Types of gamblers. - Factors influencing the onset and maintenance of GD and its features.
M3. Stimulus control and responsible return of debts.	Gambling cessation and commitment to returning debts responsibly.	- Justification of the need for this therapeutic component, and the relevance of a co-therapist. - Limiting accessibility to money, gambling venues, and gambling friends. - Commitment to accomplish stimulus control through a behavioral contract. - List of debts and returns planning.
M4. Cognitive restructuring	Identification and correction of thoughts that contribute to GD onset and maintenance.	- Explanation of the importance of thoughts and how they influence emotions, behaviors and physiological responses through the ABC model. - Definition of dysfunctional thoughts or thinking traps related to gambling. - Identification and correction of own dysfunctional thoughts.
M5. Emotion regulation	Identifying emotions and understanding its function and how to tolerate and change emotional responses.	- Understanding emotions. - Emotional avoidance and Emotion Driven Behaviors (EDBs). - Emotion regulation strategies.
M6. Planning of significant activities	Lifestyle balance.	- Planning of different significant activities according with their values (e.g., activities participants used to or already enjoy, and new activities they would like to be involved in). - Involving significant others in alternative activities.
M7. Coping skills and exposure with response prevention	Habituation to the gambling conditioned stimulus without gambling.	- Explanation of the exposition with response prevention fundaments. - Establishment of the exposition hierarchy. - Gradual exposure to different gambling-related situations according to the established hierarchy.
M8. Relapse prevention	Avoiding relapses and maintain changes gained through the intervention.	- Evaluation of the patient’s progress and achievements. - Identification of high-risk situations, and anticipation of possible breakdowns. - Review of the techniques learned to deal with these situations.

**Table 2:** SC/ERP + LBT-based ICT system usability test.

Items	First Use	After Intervention
1: I think that I would like to use this system frequently	4	4
2: I found the system unnecessarily complex	1	1
3: I thought the system was easy to use	5	5
4: I think that I would need the support of a technical person to be able to use this system	1	1
5: I found that the various functions in this system were well integrated	5	4
6: I thought that there was too much inconsistency in this system	2	1
7: I would imagine that most people would learn to use this system very quickly	5	5
8: I found the system very cumbersome to use	1	1
9: I felt very confident using the system	2	4
10: I needed to learn a lot of things before I could get going with this system	1	1

**Table 3:** SC/ERP + LBT-based ICT system usability test.

Items	First Use	After Intervention
1: I think that I would like to use this system frequently	5	5
2: I found the system unnecessarily complex	1	1
3: I thought the system was easy to use	5	5
4: I think that I would need the support of a technical person to be able to use this system	1	1
5: I found that the various functions in this system were well integrated	5	5
6: I thought that there was too much inconsistency in this system	1	1
7: I would imagine that most people would learn to use this system very quickly	5	5
8: I found the system very cumbersome to use	1	1
9: I felt very confident using the system	5	5
10: I needed to learn a lot of things before I could get going with this system	1	1

**Table 4:** Domains, categories, and illustrative ideas.

Domains	Categories (Frequency)	Illustrative Core Idea
Usefulness	Vigilance (13)	The sensation the app offers of being observed is an advantage, and it gives one the confidence to be abstinent.
	Lapse/relapse prevention (11)	Messages such as “It is not a good idea to be here” or “we recommend you leave this place” were useful to cope with gambling urges when risk situations were present and to avoid lapses.
	Stimuli control (10)	The tool helps prevent being at risk situations such as gambling-related venues.
	Accompaniment/protection (6)	It supports one throughout the intervention and protects from gambling activities.
	Reduction of the lapse/relapse duration (5)	The fact that one receives support messages for leaving the gambling activity when a lapse is produced could be useful to reduce the lapse’s duration and to avoid a relapse.
	Gambling urges habituation (1)	The app helped to stay in the gambling situation without betting until the gambling urges decreased and the ability to cope with gambling urges increased.
Improvements	Adding places to the app by contrasting the information with the co-therapist (5)	It could be interesting to contrast the information with the co-therapist about the different gambling venues patients used to go to.
	Increasing the feedback to the therapist (4)	It would be relevant for therapists to know the amount of time patients spend at every site that could be related to gambling activities in order to increase control over patients and protect them.
	Rise of the emotional impact of the messages (3)	Messages could be related to the negative consequences of gambling with a higher emotional effect in order to influence people to stop gambling when located at a gambling venue.
	Therapist assistance during a risky lapse situation (2)	The therapist could receive more information about the patient’s location for increasing support when they are in a risky situation or in the face-to-face therapy sessions.
Recommendation to other people	Extra support for other people suffering gambling problems (3)	The use of the app would be recommended to other people with gambling problems because it has several advantages (e.g., accompaniment for increasing self-efficacy to cope with gambling urges and preventing lapses).
	Assistance in the treatment of other psychological problems (1)	This tool could be useful for the treatment of other addictions, for instance, regarding cocaine, marijuana, or alcohol substances.
Safety	Confidence (8)	The app gives one the confidence that it can help because it accomplishes the function of guiding in coping with gambling problems.
	Intrusiveness (2)	The sensation of discomfort or insecurity due to aspects of threats to confidentiality using this technology only are present at the initial moment.
Usability	Ease of installation (5)	The procedure to download and install the application was easy.
	Ease of use throughout the intervention (7)	Once the app is installed and you have activated the location-based position it functions autonomously and it is easy.
Opinion for using the app after completing the intervention	Support to be abstinent (6)	The use of the app after completing the intervention, it can help cope with gambling urges and to be abstinent.
	Severity of gambling-related symptomatology (3)	Depending on the gambling severity symptoms it could be convenient to continue using the app after the intervention. In more severe cases it would be useful to remind patients when they are at risk situations and avoid lapses.
	Updating (2)	It could be convenient to use the app after the intervention updating the risky gambling-related places, because routines can change over time.

## Data Availability

For security reasons, the code of the developed platform is not publicly exposed. An updated version of the platform is available in https://symptoms.uji.es (1 March 2022).

## Supplementary Materials

The following supporting information can be downloaded at: www.mdpi.com/article/10.3390/ijerph19073769/s1. Supplementary Text S1: Geolocation System Opinion Interview (In English); Supplementary Text S2: Geolocation System Opinion Interview (In Spanish).
