# Supplementary material for: Client’s Experiences Using a Location-Based Technology ICT System during Gambling Treatments’ Crucial Components: A Qualitative Study
Source: Int J Environ Res Public Health. 2022 Mar 22;19(7):3769. doi: 10.3390/ijerph19073769 (PMC8997771; doi:10.3390/ijerph19073769)
Supplement: Supplementary file 1 [file ijerph-19-03769-s001.zip › ijerph-1648867-supplementary.pdf]

Supplementary Text S1: Geolocation System Opinion Interview

The questions that we are going to ask you below are intended to help us understand your opinion and experience regarding the geolocation system during treatment. The objective is to get a better overview of your opinion.

We would like you to answer these questions as extensively as possible. The information we extract from this interview will be used anonymously and for research purposes only.

1. After your experience, could you tell us what advantages this application has during treatment and/or what aspects could be improved?

2. After your experience, what reasons would you give when recommending or not the use of this tool to others with the same problem?

3. After your experience, why do you consider this tool to be useful or not throughout treatment?

4. After your experience, why do you consider the use of this technology to be intrusive/non-intrusive (due to aspects of threats to confidentiality)?

5. After your experience, what aspects of the tool do you think make it easier and/or more difficult to use?

6. After your experience, to what extent do you think continuing to use the tool after treatment completion could be helpful for your problem?

0 1 2 3 4 5 6 7 8 9 10

Not at all — Very much

Could you give a more extended explanation of the reasons why its use might or might not be helpful beyond treatment completion?

Supplementary Text S2: ENTREVISTA DE OPINIÓN CON EL SISTEMA DE GEOLOCALIZACIÓN

Las preguntas que vamos a hacerte a continuación pretenden ser de ayuda para conocer tu opinión y experiencia con el sistema de geolocalización durante el tratamiento. El objetivo es conocer de forma más amplia tu opinión sobre el sistema de geolocalización.

Nos gustaría que en todas las preguntas que te vamos a realizar, nos contestaras de la manera más amplia que te sea posible. La información que extraigamos de esta entrevista se empleará de manera anónima y únicamente con fines de investigación.

1. Tras tu experiencia ¿Podrías comentarnos qué ventajas habría de utilizar esta aplicación durante el tratamiento y/o qué aspectos se podrían mejorar?

2. Tras tu experiencia ¿Por qué razones recomendarías o no el uso de esta herramienta a otros con tu mismo problema?

3. Tras tu experiencia ¿Por qué razones consideras útil o no el uso de esta herramienta a lo largo del tratamiento?

4. Tras tu experiencia ¿Por qué razones consideras intrusivo/no intrusivo (por aspectos de amenazas a la confidencialidad) el uso de esta tecnología?

5. Tras tu experiencia ¿Qué aspectos de la herramienta crees que hacen que sea más fácil y/o más difícil de utilizar?

6. Tras tu experiencia ¿En qué medida crees que podría haber sido de ayuda para tu problema seguir utilizando la herramienta más allá de la finalización del tratamiento?

   0 1 2 3 4 5 6 7 8 9 10

   Nada Muchísimo

   ¿Podrías dar una explicación más extendida de cuáles serían las razones por las que podría ser de ayuda o no su uso más allá de la finalización del tratamiento?
